# Kaposi sarcoma with multiple histologic stages

**DOI:** 10.1590/0037-8682-0478-2023

**Published:** 2023-11-10

**Authors:** Felipe Tavares Rodrigues, Priscilla Filippo Alvim de Minas Santos, Maria de Fátima Guimarães Scotelaro Alves

**Affiliations:** 1 Universidade do Estado do Rio de Janeiro, Departamento de Dermatologia, Rio de Janeiro, RJ, Brasil.

**FIGURE 1:**
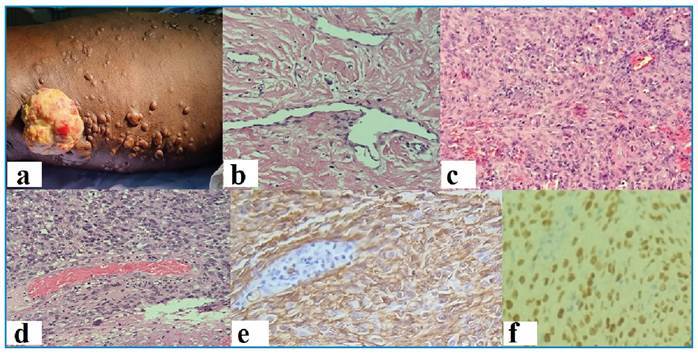
**(a)** Atypical clinical appearance: prominent tumor lesion on the thigh with satellite normochromic papules and nodules. **(b)** - Biopsy of one of the normochromic satellite papules presenting a patch stage, showing newly formed vessels protruding into a larger vascular space, which is characteristic of the promontory sign. **(c)** Plaque stage from a satellite nodule showing diffuse dermal vascular infiltrate, accompanied by increased cellularity. Notably, there are paranuclear vacuoles containing erythrocytes. **(d)** Nodular stage present in the main tumor lesion: a diffuse infiltrate of monomorphic spindled cells. **(e)** Positive immunochemistry for CD-34. **(f)** Positive immunochemistry for HHV-8.

A 42-year-old male with a history of human immunodeficiency virus (HIV) infection, a CD4 cell count of >400/μL, and an undetectable viral load for six months presented with a tumoral lesion on his lower limb accompanied by satellite normochromic nodules (Figure 1a ). A skin biopsy revealed various stages of Kaposi sarcoma (KS) (Figures 1b-1f ). The patient immigrated from Angola a year ago, where he had irregular HIV infection treatment. Following the KS diagnosis, he underwent paclitaxel treatment at an oncology center.

KS is a type of vascular sarcoma. In the 1980s, KS was one of the first opportunistic diseases reported in connection with HIV infection and was considered one of the original conditions for diagnosing AIDS. In Sub-Saharan Africa, KS is one of the most common cancers in men^1^.

Four clinical variants of this disease have been recognized: classic, endemic, and epidemic subtypes in HIV-positive patients, and the iatrogenic subtype. Type 8 human herpesvirus is the etiologic agent in all of these variants^2^. (Figure 1e)

In addition to clinical classification, typical KS is histologically categorized into three stages based on disease progression: patch, plaque, and nodular stages. During his initial examination in our service, the patient exhibited all three histologic stages simultaneously. Some parts of the tumor may have regressed due to irregular antiretroviral therapy treatment^3^.

We emphasize the rarity of the case and the importance of identifying certain histologic features, such as promontory signs, that can classify KS stages and predict the optimal treatment and management for the patient.
